# Efficacy and Safety of Aurolab Aqueous Drainage Implant Compared With Baerveldt Glaucoma Implant for Refractory Glaucoma at One Year: A Systematic Review and Meta-Analysis

**DOI:** 10.1155/2024/8617959

**Published:** 2024-11-01

**Authors:** Sandesh Raja, Umer Nisar, Owais Khan, Riteeka Kumari Bhimani, Adarsh Raja, Aayush Chaulagain

**Affiliations:** ^1^Dow Medical College, Dow University of Health Sciences, Karachi, Pakistan; ^2^Dow International Medical College, Dow University of Health Sciences, Karachi, Pakistan; ^3^Shaheed Mohtarma Benazir Bhutto Medical College Lyari, Karachi, Pakistan; ^4^Patan Academy of Health Sciences, Patan, Nepal

**Keywords:** Aurolab Aqueous Drainage Implant, Baerveldt Glaucoma Implant, glaucoma, glaucoma drainage devices

## Abstract

**Background:** Glaucoma stands as a prominent contributor to irreversible vision impairment on a global scale. For decades, the Baerveldt Glaucoma Implant (BGI) has been used to treat refractory glaucoma. Yet, the cost-effective Aurolab Aqueous Drainage Implant (AADI) has gained clinical attention as a viable alternative for managing glaucoma.

**Objective:** The purpose of this study was to evaluate and compare the efficacy and safety of AADI and BGI in the treatment of refractory glaucoma.

**Methods:** Following PRISMA guidelines, we conducted a systematic search of multiple databases, identifying relevant comparative studies assessing AADI versus BGI in patients with refractory glaucoma. Key outcomes included postoperative IOP, surgical success rates, antiglaucoma medication reduction (AGMR), and complication rates. Quality assessment was performed using the Newcastle–Ottawa Scale (NOS).

**Results:** Three studies comprised a total of 176 individuals with refractory glaucoma, with 107 patients receiving the AADI and 69 patients receiving the BGI. The meta-analysis revealed a statistically borderline significant reduction in postoperative IOP favoring the AADI at 3 months (mean difference [MD] = −2.74, *p*=0.05). There was no significant difference in the MD of AGMR between the AADI and BGI groups. The rates of total complications and surgical success did not differ significantly between the AADI and BGI groups.

**Conclusion:** AADI demonstrates promising results in reducing IOP at 3 months compared to BGI, with comparable surgical outcomes and complication rates over the long term. Further studies with larger samples are warranted to validate these findings and assess cost-effectiveness, particularly in developing countries.

## 1. Introduction

Glaucoma is a group of eye disorders associated with increased intraocular pressure (IOP) and is characterized by a gradual deterioration of retinal ganglion cells. It is distinguished by a progressive loss of peripheral vision, followed by a loss of central vision. If not treated promptly, glaucoma can result in complete blindness [1]. Glaucoma is the leading cause of blindness in the world, following cataract. In 2020, glaucoma was responsible for 11% of all global blindness in adults aged 50 and over [2]. Glaucoma drainage implant surgery involves the implantation of an artificial filtering device to reduce IOP. Glaucoma drainage devices (GDDs) are used to treat glaucoma that is at high risk of failure after trabeculectomy [3, 4]. Although patients at high risk of glaucoma face challenges, the use of aqueous drainage devices as an initial surgical treatment has shown promising results [5, 6]. To address this issue, many types of glaucoma drainage implants have been created in recent years [4, 7, 8].

GDDs include nonvalved devices, such as the Molteno implant (Molteno Ophthalmic Ltd., Dunedin, New Zealand) and the Baerveldt glaucoma implant (BGI) [Advanced Medical Optics, Inc., Santa Ana, California, USA], as well as the Aurolab Aqueous Drainage Implant (AADI) [Aurolab, Madurai, India], and valved devices such as the Ahmed glaucoma valve (AGV) [New World Medical, Rancho Cucamonga, California, USA] [9]. The BGI is a nonvalved device that is extensively used in glaucoma practice. It has a silicone plate with surface areas of 250 or 350 mm^2^. The BGI is typically located between the two rectus muscles, which are usually lateral and superior or inferior and medial. Its nonvalvular tube design helps maintain low resistance to flow, contributing to long-term reductions in IOP [10].

The AADI is a new nonvalvular GDD that is based on the structure of the BGI. It is a cost-effective alternative, particularly in developing countries with a high prevalence of pediatric glaucoma. It has a surface area of 350 mm^2^, a substantial surface area that helps to decrease IOP levels, outperforming valve-equipped devices [11].

Currently, several studies have been conducted to compare the effectiveness of the two types of glaucoma drainage implants in treating refractory glaucoma. Due to the unique advantages and disadvantages associated with each implant, there is no definitive conclusion regarding the differences in objective outcomes. To our knowledge, there has been no comprehensive assessment or publication comparing the efficiency and safety of these two approaches. In response to this gap, we conducted a meta-analysis based on existing literature to assess whether the AADI or the BGI demonstrates superior efficacy and safety in treating refractory glaucoma.

The primary aim of our study is to perform a thorough analysis of the primary outcomes, including postoperative IOP and surgical success rate, as well as secondary outcomes that encompass antiglaucoma medication reduction (AGMR) and the overall occurrence of complications.

## 2. Methods

The PRISMA recommendations, which are the preferred guidelines for publishing systematic reviews and meta-analyses, were followed in this meta-analysis [12].

### 2.1. Data Sources and Search Strategy

Up until June 2023, a complete search of the Cochrane Library, Google Scholar, Science Direct, and PubMed databases was conducted. The following combination of medical terms and keywords were used in the database searches: “glaucoma,” “high IOP,” “Aurolab aqueous drainage implant,” “AADI,” “Baerveldt glaucoma implant,” and “BGI.” A detailed search strategy is presented in the Supporting Table S1. We imposed no limitations on language, age, time, or sample size, and to find possible research that might be suitable, we carefully examined the references of the relevant literature.

### 2.2. Study Selection and Eligibility Criteria

Endnote Reference Library (Version X7.5; Clarivate Analytics, Philadelphia, Pennsylvania) was used to import each item found through a thorough search of electronic databases. Duplicates were identified and removed using this software. Subsequently, two independent reviewers (S.R. and O.K.) examined the remaining articles, initially by assessing the title and abstract, followed by a thorough evaluation of the full text. The third reviewer (A.R.) was consulted to resolve any disagreements among the reviewers. The following eligibility requirements guided the selection of the articles: (a) study type: comparative studies; (b) population: patients with refractory glaucoma (e.g., failed trabeculectomy, neovascular glaucoma, uveitic glaucoma, traumatic glaucoma, and secondary glaucoma); (c) intervention: AADI versus BGI; (d) minimum 3 months postoperative follow-up; (e) the specified outcomes of interest, including postoperative IOP, which was measured by Goldmann Applanation Tonometry; surgical success rate, defined as IOP ≥ 5 mm·Hg and ≤ 21 mm·Hg from baseline without the use of antiglaucoma medication [13]; AGMR; and total complications. The criteria for exclusion were: (a) studies not reporting the outcome of interest; (b) follow-up periods of less than 3 months; and (c) full texts without raw data.

### 2.3. Data Extraction and Quality Assessment

The following information was independently gathered by two researchers (S.R. and O.K.) from each study included in the analysis: (a) name and year of the study, (b) study design, (c) study location, (d) duration of the study, (e) number of patients in each group (AADI vs. BGI), (f) general characteristics of the patients (age, gender, and preoperative antiglaucoma medications), and (g) all the outcomes of interest.

If the data were initially provided as the median and interquartile range (IQR), our first step was to contact the authors to request the mean and standard deviation (SD) figures. However, we adopted a different approach when we did not receive a response. As a last resort, we determined the mean using the following verified formula: mean = (2*m* + *a* + *b*)/4, where “*m*” stands for the median, and “*a*” and “*b*” are the 25th and 75th percentiles, respectively [14]. We used the Cochrane Collaboration's formula to calculate the SD: IQR = 1.35 × SD [15].

The NOS [16], which rates research on a scale from 0 to 9, was used to evaluate the methodological quality of the cohort and case-control retrospective investigations. Three criteria were used to assess the studies: selection, comparability, and outcome. A grade of 5 stars for research quality denoted a relatively high standard. All items were independently evaluated by two researchers (U.N. and O.K.), and any discrepancies were resolved by consensus. A third researcher (S.R.) served as an arbitrator when necessary.

### 2.4. Statistical Analysis

Review Manager (RevMan) version 5.4.1 was used for this meta-analysis in accordance with The Cochrane Collaboration's (2020) recommendations. For pooling categorical outcomes, risk ratios (RRs) and their accompanying 95% confidence intervals (CIs) were calculated using a random-effects meta-analysis methodology. For continuous outcomes, a random-effects meta-analysis was also performed to determine mean differences (MD) and their accompanying 95% CIs. The combined findings are depicted in the form of forest plots. We measured the degree of heterogeneity among the studies using Higgins *I*^2^, where values between 25% and 50% were considered mild, 50%–75% were considered moderate, and values exceeding 75% were considered severe heterogeneity [17].

## 3. Results

### 3.1. Study Selection and Characteristics

**Figure 1:**
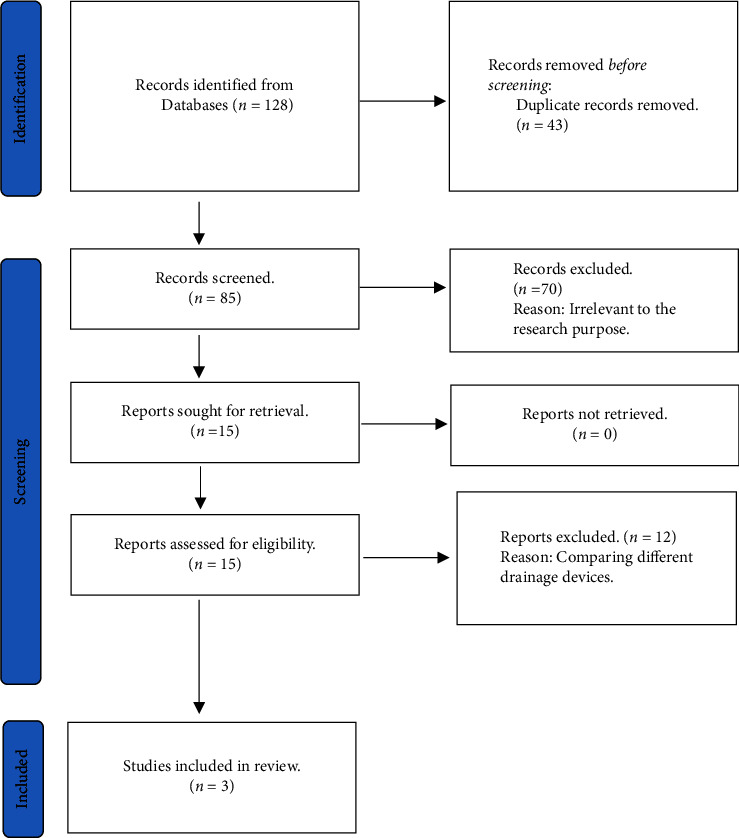
PRISMA flowchart.

Our comprehensive literature search yielded 128 relevant articles, from which 43 duplicates were identified and removed. Subsequent screening of the remaining articles determined that 70 were irrelevant to the research objective, as they did not compare the two drainage devices. Additionally, 12 studies were excluded because they compared different drainage devices. The PRISMA flowchart shows a summary of our findings from our extensive literature review (Figure 1). A total of 3 studies [18–20] were included in this meta-analysis, comprising a total of 176 patients with glaucoma, with 107 patients receiving AADI and 69 patients receiving BGI, all of which fulfilled the inclusion and exclusion criteria. The mean follow-up time was 17.0 months. The research by Aljaloud et al. [19] evaluated the efficacy of both standard and trimmed AADIs (with the lateral wings of the trimmed implant modified to achieve a plate size approximately equivalent to a Baerveldt-250 implant) in comparison to the BGI, although only data for the standard AADI was used for analysis. Rateb et al. [18] did not report the SD for IOP; therefore, their study was not included in the IOP analysis. General characteristics of the studies included in the analysis are presented in Table 1. Baseline characteristics of the patients in each study are provided in Table 2.

### 3.2. Quality Assessment of Included Studies

Quality assessment for the included observational studies was done using NOS, as two of the studies were retrospective cohort [18, 19] and one was a case-control study [20]. All the included studies were of high quality. Detailed quality assessment is provided in Supporting Table S2 for the retrospective cohort studies and Supporting Table S3 for the retrospective case-control study.

### 3.3. Primary Outcomes

#### 3.3.1. Postoperative IOP

**Figure 2:**
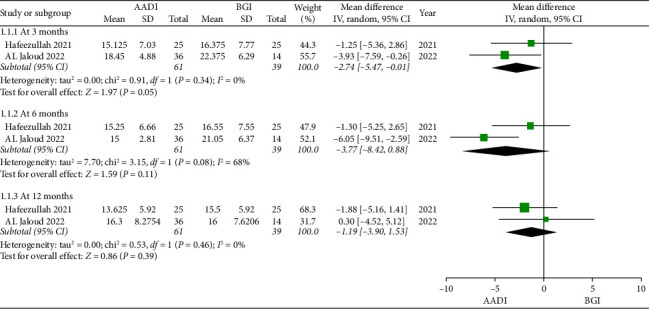
Forest plot of overall postoperative IOP.

This meta-analysis, using a random-effects model and incorporating data from two studies, demonstrated a statistically borderline significant reduction in postoperative IOP in patients from the AADI group compared to the BGI group at 3 months (MD: −2.74, 95% CI [−5.47, −0.01]; *p*=0.05; *I*^2^ = 0%), while at 6 months, the decrease was not statistically significant (MD: −3.77, 95% CI [−8.42, 0.88]; *p*=0.11; *I*^2^ = 68%). By the 12-month follow-up, no significant difference in postoperative IOPs was observed between the two groups (MD = −1.19, 95% CI [−3.90, 1.53]; *p*=0.39; *I*^2^ = 0%) (Figure 2).

#### 3.3.2. Surgical Success Rate

**Figure 3:**
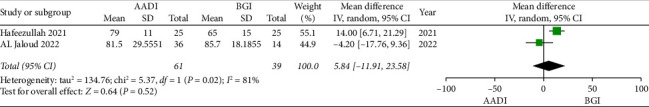
Forest plot of surgical success rate.

A random-effects model, incorporating data from two studies, indicated a statistically nonsignificant difference in surgical success rates among patients in the AADI group compared to those in the BGI group (MD = 5.84, 95% CI [−11.91, 23.58]; *p*=0.52; *I*^2^ = 81%) (Figure 3).

### 3.4. Secondary Outcomes

#### 3.4.1. Total Complications

**Figure 4:**
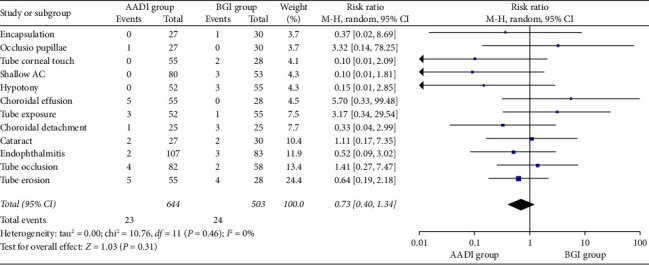
Forest plot of total complications.

Total complications comparing the two groups were described in the included studies. The incidence of encapsulation (pooled RR: 0.37, 0.02–8.69), occlusio pupillae (pooled RR: 3.32, 0.14–78.25), tube corneal touch (pooled RR: 0.10, 0.01–2.09), shallow AC (pooled RR: 0.10, 0.01–1.81), hypotony (pooled RR: 0.15, 0.01–2.85), choroidal effusion (pooled RR: 5.70, 0.33–99.48), tube exposure (pooled RR: 3.17, 0.34–29.54), choroidal detachment (pooled RR: 0.33, 0.04–2.99), cataract (pooled RR: 1.11, 0.17–7.35), endophthalmitis (pooled RR: 0.52, 0.09–3.02), tube occlusion (pooled RR: 1.41, 0.27–7.47), and tube erosion (pooled RR: 0.64, 0.19–2.18) were comparable in the AADI and BGI groups. However, pooled results showed similar overall complication incidence between the two groups [RR = 0.73, 95% CI (0.40, 1.34), *p*=0.31]. There was no significant heterogeneity observed [*p*=0.46; *I*^2^ = 0%] (Figure 4).

#### 3.4.2. AGMR at 1 Year

**Figure 5:**
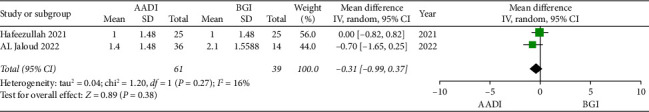
Forest plot of reduction in antiglaucoma medication at 1 year.

Two studies reported the use of antiglaucoma medication postoperatively. A random-effects model was used to pool the combined effect, which showed that there was no significant difference in postoperative AGMR [MD = −0.31, 95% CI (−0.99, 0.37), *p*=0.38]. There was no significant heterogeneity observed among the studies [*p*=0.27; *I*^2^ = 16%] (Figure 5).

## 4. Discussion

In the present meta-analysis, a thorough assessment was conducted to evaluate the utilization of AADI and BGI in cases of refractory glaucoma. The use of glaucoma drainage implants has spanned over 2 decades, and the effectiveness of glaucoma surgery for refractory glaucoma may be influenced by various types of drainage implants. Although AADI is manufactured to adhere to the specifications of BGI 350 mm^2^ and serve as valveless drainage devices, the primary differentiation lies in the composition process of AADI. Subtle discrepancies in the medical-grade silicone and the quantity of barium used slightly deviate from those employed in BGI. Consequently, this variation yields a device characterized by enhanced flexibility [21]. However, the analysis did not reveal compelling evidence to support the notion that AADI, compared to BGI, is associated with diminished postoperative IOP at the 1-year mark, a reduction in the use of antiglaucoma medications, or a decrease in overall complications.

The study by Hong et al. [22] conducted a meta-analysis to compare AADI with AGV for the treatment of refractory glaucoma. The findings demonstrate that AADI is superior in both reducing IOP and AGMR compared to AGV, with a higher surgical success rate. This difference may be attributed to two factors: the larger surface area of AADI, which enhances its effectiveness in lowering IOP, and the absence of valve-induced resistance compared to the valved AGV, promoting more efficient aqueous humor flow to the plate [23]. Both implants exhibit similar rates of adverse events; however, AADI is associated with more instances of choroidal detachment due to lower postoperative pressures. This may be attributed to the AGV's restrictive valve, which prevents hypotony, while the AADI lacks a flow restrictor, theoretically resulting in more frequent hypotony and its associated complications [24].

In comparison, our study examining AADI versus BGI found that AADI demonstrated a borderline significant reduction in IOP at 3 months, although no differences were observed at 6 and 12 months. The AGMR postoperatively was not significantly different between AADI and BGI. Regarding surgical success rates, while there was no statistically significant difference in our study, both implants exhibited trends toward comparable outcomes. Both studies reported comparable adverse event rates, with no significant complications unique to either implant. The lack of significant difference in outcomes between AADI and BGI can be attributed to the fact that both implants operate on the same underlying principle of aqueous drainage, which may yield similar effectiveness. Additionally, the borderline significant difference observed in IOP reduction could be explained by the variations in implant size; Aljaloud et al. [19] utilized a BGI with a surface area of 250 mm^2^, whereas AADI implants typically have a larger surface area of 350 mm^2^, potentially influencing the comparative effectiveness.

This investigation underscores the potential impact of divergent plate sizes on the efficacy of AADI in reducing postoperative IOP. A recent study conducted by Puthuran et al. [25], shows that the AADI group is better at reducing IOP at the 3-month interval, which is also supported by our results. However, there was no significant difference in IOP reduction at 12 months between either of the groups, suggesting that both methods are equally effective at reducing IOP at that time.

The surgical success rate is defined by a reduction in IOP without the need for additional antiglaucoma medications. Our results showed similar outcomes in both groups, suggesting that neither group had a higher success rate than the other. The reduction in antiglaucoma medication usage was assessed between the AADI and BGI groups. Our analysis revealed comparable outcomes in medication reduction between the two groups, indicating no significant difference in medication reduction rates. These similarities could be explained by the fact that both implants function according to similar principles.

The likelihood of corneal edema is highest in postoperative patients undergoing BGI, while tube-related problems, such as tube blockage, tube erosion, and tube malposition, are the second most frequent complications [26]. The bleb is not directly exposed to the inflammatory mediators from the anterior chamber; therefore, its chances of encapsulation are significantly reduced in patients who underwent AADI [21]. Despite previous research indicating that AADI is less likely to cause complications, our analysis found no significant difference in the complications experienced by patients who underwent AADI or BGI [27].

According to our research, the results indicate that AADI showed superior efficacy in reducing postoperative IOP compared to BGI at 3 months. Additionally, we found no significant disparities in the overall occurrence of complications between AADI and BGI. Given its higher effectiveness in reducing IOP, AADI can be considered a viable alternative to BGI. Moreover, AADI's affordability makes it particularly suitable for countries with limited resources as a potential treatment option for glaucoma [28].

The observed high heterogeneity in several outcomes may be attributed to multiple factors. First, the use of plates of different sizes across the studies could influence the results, as variations in surface area may affect IOP dynamics. Additionally, the lack of standardized reporting on examination methods, including those for visual field assessments, anterior segment evaluations, and fundus examinations, adds complexity to the interpretation of outcomes. These inconsistencies in methodology may lead to variability in the reported results, ultimately contributing to the high heterogeneity observed in this meta-analysis.

The limitations identified within this meta-analysis encompass several aspects. Firstly, the inclusion of observational studies may introduce potential bias into the findings. Additionally, the limited number of studies and their relatively small sample sizes pose limitations on the generalizability of the results. While AADI presents as a cost-effective option, particularly beneficial for developing countries, the absence of pertinent cost data precludes an analysis of cost-effectiveness. Furthermore, the use of plates of different sizes and the lack of standardized reporting on examination methods, including those for visual field, anterior segment, and fundus evaluations, across the included studies adds complexity to the interpretation of outcomes, as varying examination techniques could potentially influence results. Moreover, the meta-analysis does not explicitly address patients' comorbidities, such as diabetes, hypertension, cardiovascular diseases, and autoimmune disorders, which could significantly impact surgical outcomes. The inclusion of children in one of the studies raises considerations regarding age group differences. Additionally, the use of different plate sizes could have affected postoperative IOP. This underscores the necessity for further research with larger sample sizes and comprehensive considerations to gain deeper insights into the preference for either implant.

## 5. Conclusion

Our meta-analysis examined the effectiveness of AADI and BGI in managing refractory glaucoma. While AADI demonstrated borderline significant reductions in IOP at 3 months, no notable differences were observed compared to BGI at the 6-month and 1-year marks. Surgical success rates, complications, and medication reductions were comparable between the two implants, likely due to their similar operational characteristics. However, we acknowledge limitations such as the inclusion of observational studies, small sample sizes, and study heterogeneity.

## Figures and Tables

**Table 1:** General characteristics of studies included in meta-analysis.

Studies	Design	Year	Country	Study duration	Device type	
					AADI group	BGI group
Rateb et al.	Retrospective cohort	2019	Egypt	April 2016 to May 2018	AADI	BGI, Ns
Hafeezullah et al.	Retrospective case control	2021	Saudi Arabia	May 2015 and May 2017	AADI	350 mm^2^ BGI
Al Jaloud et al.	Retrospective cohort	2022	Saudi Arabia	April 2016 to December 2018	AADI	250 mm^2^ BGI

Abbreviations: AADI, Aurolab Aqueous Drainage Implant; BGI, Baerveldt Glaucoma Implant; Ns, nonspecific.

**Table 2:** Baseline characteristics table of patients in the included studies.

Table S2B: Baseline characteristics between AADI and BGI of included studies in the meta-analysis												
Studies	Sample size	AADI group	BGI group	Gender		Pre IOP in (m ± SD)		Pre AGM in (m ± SD) and (M (IQR))		Follow up (in months)	Age	
				AADI group	BGI group						AADI group	BGI group
				Male\female	Male\female	AADI group	BGI group	AADI group	BGI group			
Rateb et al.	57	27	30	17\10	16\14	34 ± 5	29 ± 2.2	3.2 + 0.6	3.1	1 week and 3 and 6 months	28.12 months	34.24 months
Hafeezullah et al.	50	25	25	17\8	11\14	26.25 ± 10.37	31.75 ± 11.11	3.0 (1.0)	3.0 (2.0)	21.1 ± 10.0 months	32.2 ± 24.6 years	32.0 ± 24.5 years
Al Jaloud et al.	50	36	14	23\13	7\7	26.7 ± 9.7	30.7 ± 9.1	3.4 ± 1.2	3.8 ± 0.7	24 months	34.2 ± 23.8 years	34.0 ± 26.7 years

Abbreviations: AADI, Aurolab Aqueous Drainage Implant; BGI, Baerveldt Glaucoma Implant; (M (IQR)), median and interquartile range; (m ± SD), mean and standard deviation; Pre AGM, preoperative antiglaucoma medication; Pre IOP, preoperative intraocular pressure.

## Data Availability

The dataset that supports the findings of this article is included within the manuscript/supporting file.

## Supporting Information

Supporting Table 1—Search Strategy of this meta-analysis. Supporting Table 2—Newcastle–Ottawa quality Assessment scale for retrospective cohort studies. Supporting Table 3—Newcastle–Ottawa quality Assessment scale for retrospective case control study.
