# Supplementary material for: Efficacy and Safety of Aurolab Aqueous Drainage Implant Compared With Baerveldt Glaucoma Implant for Refractory Glaucoma at One Year: A Systematic Review and Meta-Analysis
Source: J Ophthalmol. 2024 Nov 1;2024:8617959. doi: 10.1155/2024/8617959 (PMC11548946; doi:10.1155/2024/8617959)
Supplement: Supporting Information — Additional supporting information can be found online in the Supporting Information section. [file 8617959.f1.docx]

**Supplementary Material**

Supplementary Table 1 – Search Strategy of this meta-analysis

Supplementary Table 2 –Newcastle-Ottawa quality Assessment scale for retrospective cohort studies

Supplementary Table 3 – Newcastle-Ottawa quality Assessment scale for retrospective case control study

**Supplementary Material**

Supplementary Table 1 – Search Strategy of this meta-analysis

| Search strategy for databases. |  |
| --- | --- |
| (Aurolab aqueous drainage implant OR AADI OR Aurolab implant) AND (Baerveldt implant OR BGI OR Glaucoma drainage device OR aqueous shunt device) AND (Glaucoma OR High intra-ocular pressure OR high IOP OR Ocular hypertension) |  |
|  |  |
|  |  |
|  |  |

Supplementary Table 2 –Newcastle-Ottawa quality Assessment scale for retrospective cohort studies

|  | Study name | |
| --- | --- | --- |
|  | Rateb 2019 | Al Jaloud 2022 |
| **Selection (4)** |  |  |
| Representativeness of the exposed cohort | * | * |
| Selection of the non-exposed cohort | * | * |
| Ascertainment of exposure | * | * |
| Demonstration that outcome of interest was not present at start of study | * | * |
| **Comparability (2)** |  |  |
| Comparability of cohorts on the basis of the design or analysis | ** | ** |
| **Outcome (3)** |  |  |
| Assessment of outcome | * | * |
| Was follow-up long enough for outcomes to occur | * | * |
| Adequacy of follow up of cohorts | * | * |
| **Total (9)** | **9** | **9** |

Supplementary Table 3 – Newcastle-Ottawa quality Assessment scale for retrospective case control study

|  | Study name |
| --- | --- |
|  | Hafeezullah 2021 |
| **Selection (4)** |  |
| Is the case definition adequate? | * |
| Representativeness of the cases | * |
| Selection of Controls | * |
| Definition of Controls | * |
| **Comparability (2)** |  |
| Comparability of cases and controls on the basis of the design or analysis | ** |
| **Exposure (3)** |  |
| Ascertainment of exposure | * |
| Same method of ascertainment for cases and controls | * |
| Non-Response rate | * |
| **Total (9)** | **9** |
